# Epidemiological Characteristics and Role of Connexin-43 in Patients of Syndactyly Attending a Tertiary Care Center in Odisha, India

**DOI:** 10.7759/cureus.30327

**Published:** 2022-10-15

**Authors:** Sanjukta Sahoo, Suranjana Banik, Sanjay K Giri, Prabhas R Tripathy, KC Pradheep Kumar

**Affiliations:** 1 Anatomy, All India Institute of Medical Sciences, Bhubaneswar, Bhubaneswar, IND; 2 Burns and Plastic Surgery, All India Institute of Medical Sciences, Bhubaneswar, Bhubaneswar, IND

**Keywords:** reconstruction, plastic surgery, connexin 43, epidemiology, syndactyly

## Abstract

Background: Syndactyly is one of the most common hereditary limb malformations. Yet, epidemiological data in our state is not reported. The current study aims to understand the Connexin-43 expression in such patients.

Methodology: A retrospective cohort study was done in the Department of Plastic surgery and Anatomy of All India Institute of Medical Sciences (AIIMS), Bhubaneswar. The study duration was three years, between 2019 and 2022. The total number of cases was 49 and included patients diagnosed with Syndactyly seeking surgical intervention. The demographic details and the personal and disease history were collected, analyzed, and interpreted. Immunohistochemistry study using Connexin-43.

Results: Out of the 49 patients, 26 (53.1%) were male, and 23 (46.9%) were female. Thirty nine (79.6%) had syndactyly, and 10 (20.4%) were diagnosed with syndactyly associated with another syndrome. Both complete and incomplete syndactyly was found. Strong membranous positivity of Connexin-43 was found in the keratinocytes of the stratum spinosum layer of the epidermis, while the stratum granulosum and stratum basale layer revealed negative staining.

Conclusions: Syndactyly is mostly non-familial, sporadic with male preponderance affecting unilaterally and in incomplete form in our geographical location. We found an overt expression of Connexin-43 in these patients' stratum basale.

## Introduction

Syndactyly is one of the most common hereditary limb malformations depicting a prevalence of 3-10 per 10,000 births and a higher prevalence of one per 2,000 or 3,000 live births. Syndactyly originating from the Greek words (Syn meaning together and Dactylos meaning digits) is a digital malformation of the digits in which adjacent fingers of hands and/or toes are webbed in appearance [1,2]. Due to high phenotypic variation and genetic diversity, a single reason cannot be attributed to the occurrence of syndactyly [2]. Earlier, it was considered that syndactyly could be only congenital [3], but, with the evolution of molecular studies on embryology and morphogenesis, it has been found that syndactyly can be primary when the defect occurs in-utero, or it can be secondary, where adhesion between adjacent raw surfaces which were not previously joined might happen [1]. Primary syndactyly may be De-novo or due to mutations, whereas secondary is generally due to defects in healing or reconstructions [4].

Primary syndactyly cases show autosomal dominant inheritance. Heterogeneous mutations of the *HOXD13 *gene have been found to be associated with syndactyly [5]. Penetrance is variable but can be prominent for over seven generations, with more severe expansions causing widespread involvement of digits [6]. Connexin-43 (Cx43), a gap junction protein, maintains gap junction intercellular communication, and this, in turn, regulates osteoblast formation, differentiation, survival, and apoptosis and mediates web space formation. Any defect in this protein signaling can lead to a defect in the development of digits [7]. Although connexin's exact role in skin development is not well known, a mutation in Cx43 results in some skin disorders, emphasizing that Cx43 is essential for normal skin development [8]. The current study aims to understand the role of Cx43 expression in skin development along with the epidemiological patterns of syndactyly.

## Materials and methods

Methods

Study Design

It was a retrospective cohort study.

Study Setting

Department of Burns and Plastic surgery and Anatomy of All India Institute of Medical Sciences (AIIMS) Bhubaneswar.

Study Duration

The time period was between November 2019 and May 2022.

Study Participant

Forty-nine patients were included with the diagnosis of syndactyly seeking surgical intervention and included all age groups and gender. The sampling technique was convenient sampling.

Sample Collection

The demographic details and the personal and disease history were collected, and entry was made into an excel sheet (Microsoft Windows 10 version) after taking proper consent from the ethical committee. The clinical data recorded were the digits involved, the level of web involvement, measurements of fingers, and the appearance of the fingernails. The cranium, face, torso, and lower extremities were examined for any other congenital anomaly. The type of surgery done for the patients was also recorded. The resected skin sample (minimum length and thickness were 2 cm) obtained during corrective surgery which was collected and stored in normal buffer formalin was used for the IHC study. The samples were subjected to a biopsy, hematoxylin & eosin (H&E) staining immunohistochemistry (IHC) study for expressing Cx43 in the anatomy department. Excised skin from five patients undergoing procedures, other than syndactyly, acted as a control for our cases and were also studied for H&E and the same IHC marker. The expression in the keratinocytes was analyzed according to the intensity and location of the staining using the Applied Spectral Imaging Software of the Olympus BX63 microscope.

Statistical analysis

The data were subjected to statistical analysis using Statistical Package for Social Sciences (SPSS) 26 (IBM Corp., Armonk, NY) and the result was generated using mean standard deviation. The difference in mean between the two groups was assessed by independent sample t-test or Mann-Whitney U test. Pearson's or Spearman's correlation coefficients determined the correlation between two quantitative variables. The normality of the variables was checked using the Kolmogorov-Smirnov test. A P-value less than 0.05 was considered statistically significant.

IHC Interpretation

The IHC scoring was done per the software guidelines of the applied spectral imaging (ASI) platform of the Olympus BX 63 intelligent microscope.

Ethical Clearance

Institutional ethical approval was taken from AIIMS Bhubaneswar for the study (T/IM-NF/Anatomy/19/25).

## Results

Epidemiology

**Figure 1:**
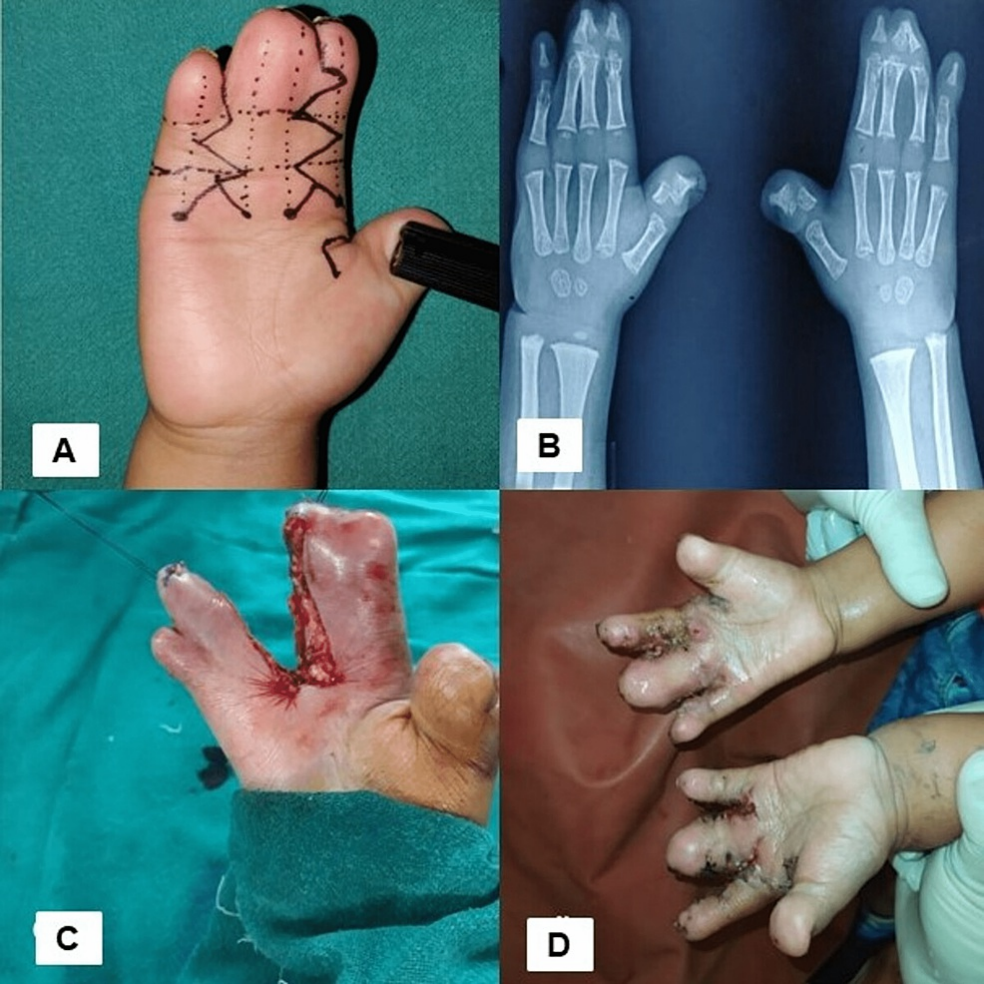
Images showing (a) planning and release of second web space complete syndactyly and 4th webspace incomplete syndactyly. (b) X-ray showing fusion of third and fourth distal phalanx in syndactyly. (c) Intraoperative after separation of digits. (d) Postoperative follow-up case.

Among the total 49 patients, there were 26 (53%) male and 23 (47%) female patients. Forty four (90%) had no family history of syndactyly. A majority belonged to the age group <10 years, with 80% having isolated syndactyly (not associated with syndromes). Twenty seven (55.1%) of patients had unilateral involvement and an incomplete syndactyly pattern. 61.2 % were treated with only a simple release that required a split skin graft split-thickness skin graft (SSG), 17 (34.8%) with a release (straight cut of web spaces and defect created) + full thickness split graft (FTSG), and one (2%) had a release (straight cut of web spaces and defect created) + K-wire fixation and Release (straight cut of web spaces and defect created) + Flap (local square flap) shown in Figures 1a-1c.

More than half of the patients, 27 (55.1%), had unilateral, and 22 (44.9%) had bilateral. The details of the demography are shown in Table 1.

**Table 1:** Epidemiological data of the patients with syndactyly

Variables		Frequency (n)	Percentage (%)
Age in years	0-10	31	63.3
	11-20	12	24.5
	21-30	3	6.1
	> 30	3	6.1
Sex	Male	26	53.1
	Female	23	46.9
Family History	Yes	5	10.2
	No	44	89.8
Type of syndactyly	Syndactyly	39	79.6
	Syndactyly associated with a syndrome	10	20.4
Dexterity	Unilateral	27	55.1
	Bilateral	22	44.9
Pattern of syndactyly	Complete	22	44.9
	Incomplete	27	55.1
Web space involvement	1	1	2
	2	5	10.2
	3	15	30.6
	4	3	6.2
	Multiple	25	51
Type of surgical procedure	Release + SSG	30	61.2
	Release + FTSG	17	34.8
	Release + K-WIRE FIXATION	1	2
	Release + FLAP	1	2

Statistical analysis using the Chi-square test between dexterity and the association of syndrome was 6.19 (p-value = 0.013). Similarly, the Chi-square value for the pattern and association of the syndrome was 10.3 (p-value = 0.001). 

Histopathology

The sections from the lesion of syndactyly showed hyperkeratosis, orthokeratosis, irregular acanthosis, and elongated rete ridges. Appendageal structures are not seen in any of the biopsies. Subcutis seems normal. 

IHC interpretation of Cx43 in syndactyly lesion

All five control samples showed strong membranous positivity for Cx43 in the stratum basale of the epidermis and only mild/focal positivity in the stratum spinosum layer of the epidermis. The keratinocytes in the stratum granulosum layer were interpreted as negative. However, the syndactyly cases showed strong membranous positivity in the keratinocytes of the stratum spinosum layer of the epidermis, while the stratum granulosum and stratum basale layer revealed negative staining.

**Figure 2:**
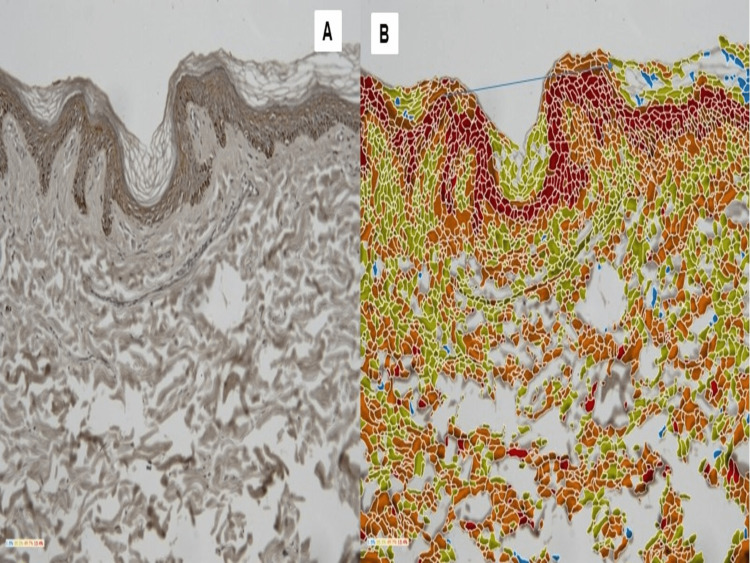
Microscopic picture of a control showing positivity in the keratinocytes of stratum spinosum layer of the epidermis, while stratum granulosum and stratum basale layer revealed a negative staining in raw image (a) and software-processed image (b).

**Figure 3:**
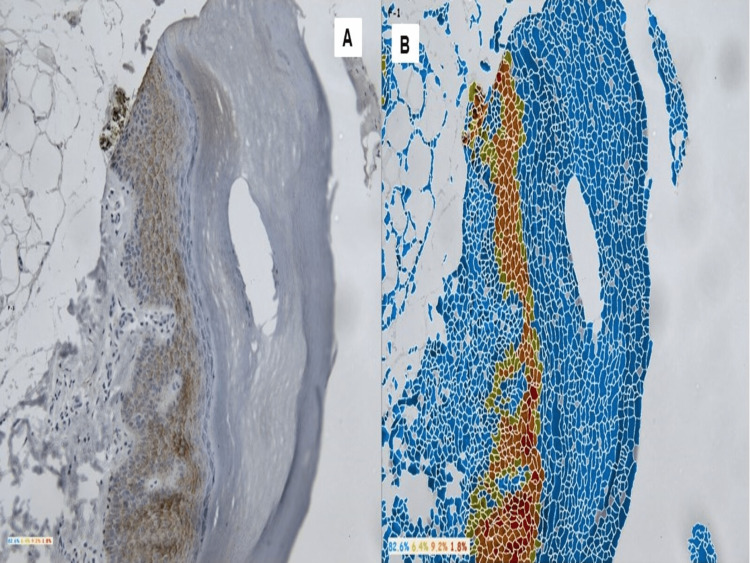
Macroscopic picture of a control showing- strong positivity for Cx43 in the stratum basale of the epidermis in raw image (a). Software-processed image of control showing the highly expressive cells are denoted by red color comprising of 13.4%. The moderate expression was denoted by orange followed by green comprising of 49.7% and 35.3%, respectively (b).

The expression of positivity was shown by color coding of blue, green, orange, and red. The highly expressive cells were denoted by red color (Grade +3), the moderate expression was denoted by orange followed by green (Grade 1-2), and the negative expression was denoted by blue (Grade 0). This is illustrated in Figures 2a, 2b, 3a, 3b.

## Discussion

There are varying reports of global syndactyly incidence in the literature, and in the present study, the majority of cases had isolated syndactyly with male preponderance. An average of 5.4% of patients get syndactyly surgery in New York, and the surgery takes place at an early age, with few needing skin grafting [9]. In the present study, too, the most common age of presentation was early. Symptoms presented within 0-10 years of age. In most studies, surgical release is the recommended treatment for syndactyly, and re-operation was also required in many cases [10-12]. In our country, the rates of surgery could not be compared to other populations due to a paucity of studies. However, all the patients who were included in this study were managed surgically involving release with SSG, Release with FTSG, Release with K-Wire Fixation, and Release with Flap.

In the present study, most patients had no family history of syndactyly, pointing towards its sporadic nature. This justifies the theory that even if no genetic predisposition exists, the environmental factors in-utero might predispose the fetus to syndactyly and other congenital hand abnormalities [13]. Syndactyly is documented to be autosomal dominant with variable expression and penetrance. It is found to have reduced penetrance in the females, thus having more male dominance (53.1%). Though in many studies, syndactyly has been found to be associated with multiple syndromes [13], the present study had normal karyotypes. Unilateral and incomplete syndactyly dominated our findings with 55.1% of the cases, and most (51%) had multiple web spaces involved.

The Connexin family has six types, which play an essential role in forming gap junctions allowing passage of small molecules and ions, and Cx43 is expressed in the developing limb bud with the patterning of digits and cartilage condensation [14]. The Cx43 gene has been determined in the human chromosome 6 sequence. In mouse embryogenesis, Cx43 is linked at the blastocyst stage and involved in various aspects of organogenesis. Thus, its altered expression has been associated with developmental anomalies involving the branchial arches, optic vesicles, neural crest defects, and sclerotomes. In humans, their expression has not been extensively studied. A mutation in the Cx43 has been reported in oculodentodigital dysplasia in humans by Van Es et al. [15]. Recently, Zhang et al. have found Cx43 to be ubiquitously expressed in the skin by all types of skin cells, such as keratinocytes, fibroblasts, endothelial and basal cells, melanocytes and dermal papilla cells. Some evidence suggests that Cx43 plays a vital role in skin repair, development, and tumor and metastasis [16]. Their study in syndactyly has not been previously investigated. We also found a strong positive result for the protein expression in the stratum spinosum layer of the normal skin (control in our study), which was very mild in intensity, in concordance with a study by Richard [17]. We also observed a strong continuous expression of the protein Cx43 in the epidermis stratum basale in our all-control samples, unlike the study by Richard [17], as they found a patchy distribution of the protein in the stratum basale. In the present study, the lesioned skin of syndactyly showed a strong expression of Cx43 in the stratum spinosum, while negative expression is observed in the stratum granulosum and stratum basale.

Our observations are novel but may need more extensive similar studies to support the involvement of the Gap-junction protein, Cx43, in syndactyly. We also acknowledge that our control sample was small due to difficulty getting normal excised skin. We also have not done karyotyping in our study. Nevertheless, it opens a new horizon for future investigation in this direction.

The limitation of the study is that it is not a representative study of the entire geographical area. The study when including different ethnic groups and in larger sample size can give a more accurate representation. The study can be repeated with more immunohistochemical markers and not a single one. The syndromes associated with syndactyly need more attention in a larger cohort.

## Conclusions

Syndactyly is mostly non-familial, sporadic with male preponderance in our geographical location. It mostly affects children <10 years, is unilateral in occurrence, isolated in presentation and most commonly in the incomplete form.

We found an overt expression of Cx43 in these patients' stratum spinosum layer of the epidermis of the skin in comparison to normal people who showed positivity for Cx43 in the stratum basale of the epidermis and only mild/focal positivity in the stratum spinosum. The gender prevalence, as well as the pattern of expression of Cx43, should be studied in a larger population along with the study of different mutations which can unfold unknown facts about Syndactyly and the associated clinical heterogenicity. This can facilitate surgical procedures and expected outcomes. Also, molecular mechanisms in non-syndromic Syndactyly can explain variable pathogenesis and facilitate genetic counseling.
